# YoDe-Segmentation: automated noise-free retrieval of molecular structures from scientific publications

**DOI:** 10.1186/s13321-023-00783-z

**Published:** 2023-11-20

**Authors:** Chong Zhou, Wei Liu, Xiyue Song, Mengling Yang, Xiaowang Peng

**Affiliations:** School of Informatics, Hunan University of Chinese Medicine, Changsha, 410208 Hunan People’s Republic of China

**Keywords:** Deep learning, Image segmentation, Molecular structure detection, Optical chemical structure recognition

## Abstract

In chemistry-related disciplines, a vast repository of molecular structural data has been documented in scientific publications but remains inaccessible to computational analyses owing to its non-machine-readable format. Optical chemical structure recognition (OCSR) addresses this gap by converting images of chemical molecular structures into a format accessible to computers and convenient for storage, paving the way for further analyses and studies on chemical information. A pivotal initial step in OCSR is automating the noise-free extraction of molecular descriptions from literature. Despite efforts utilising rule-based and deep learning approaches for the extraction process, the accuracy achieved to date is unsatisfactory. To address this issue, we introduce a deep learning model named YoDe-Segmentation in this study, engineered for the automated retrieval of molecular structures from scientific documents. This model operates via a three-stage process encompassing detection, mask generation, and calculation. Initially, it identifies and isolates molecular structures during the detection phase. Subsequently, mask maps are created based on these isolated structures in the mask generation stage. In the final calculation stage, refined and separated mask maps are combined with the isolated molecular structure images, resulting in the acquisition of pure molecular structures. Our model underwent rigorous testing using texts from multiple chemistry-centric journals, with the outcomes subjected to manual validation. The results revealed the superior performance of YoDe-Segmentation compared to alternative algorithms, documenting an average extraction efficiency of 97.62%. This outcome not only highlights the robustness and reliability of the model but also suggests its applicability on a broad scale.

## Introduction

A significant amount of molecular structure data is embedded within chemistry-centric literature. However, this valuable information remains largely untapped owing to inadequate curatorial practices and the absence of comprehensive open-access repositories [1]. Furthermore, the inherent format of these molecular structures does not lend itself to straightforward computer interpretation [2]. Consequently, data scientists are frequently tasked with the manual extraction of these molecular structures from scientific texts—a process that, when manually executed, is both labour-intensive and susceptible to inaccuracies [3]. This underscores the necessity for automated, precision-focused extraction methods, essential for reintroducing and enhancing the accessibility of chemical data in open-access repositories. The retrieval of molecular structures from academic papers is the foundational procedure in optical chemical structure recognition (OCSR) [4]. Once extracted, these 2D molecular structure visuals can be seamlessly transitioned into simplified molecular-input line-entry system (SMILES) representations [5], facilitating scientific inquiry.

The field of OCSR has witnessed substantial evolution over the past decades, with a growing emphasis on harnessing deep learning. These methodologies can effectively identify atoms and bonds in pristine chemical molecular structure illustrations, enabling the reconstruction of chemical molecular structures or their direct transmutation into encoding formats like SMILES or DeepSMILES [6–11]. However, noise-free molecular structures are not always readily available. These need to be often derived from academic literature or 2D images to obtain complete, noise-free chemical molecular structure descriptors. Notably, efforts toward molecular structure extraction do not reflect the progress made in molecular structure recognition, and research in the former area is limited.

Recently, studies focusing on deriving complete, noise-free molecular structure descriptions from texts or 2D images have been classified into two primary categories. The first employs rule-based techniques, targeting the extraction of molecular descriptors from 2D visuals, whereas the latter is anchored in deep learning to extract molecular structures. Herein, we present an overview of the molecular structure segmentation algorithms that have gained prominence in contemporary research. The second phase in the optical structure recognition application (OSRA) involves segmenting molecular structures. This segmentation process determines molecular structures based on the dimensions of the rectangular bounding box surrounding the pertinent region, combined with the ratio of black to white pixels within this box [12]. Another tool, ChemSchematicResolver (CSR), can efficiently segment images that contain only labels and molecular structures [13]. Both of these segmentation techniques are predicated on rule-based algorithms.

In a 2019 study, Staker et al. introduced a deep-learning-oriented OCSR tool [14], harnessing the capabilities of U-Net [15] for molecular structure segmentation. This model estimates the likelihood of each pixel in an image being part of a particular structure. Pixels predicted to be part of the molecular structure are subsequently masked, ensuring the segmentation of the entire molecular structure.

In 2021, Rajan et al. presented a deep-learning-centric OCSR tool named DECIMER, encompassing a molecular structure segmentation module called DECIMER Segmentation [16]. The foundation of this program is the Mask R-CNN [17] framework, paired with a molecular structure detection algorithm. DECIMER Segmentation first recognises segments of the molecular structure and masks these identified regions by scanning the entire document page. Subsequently, this mask is augmented via pixel seeding, masking the entire molecular region. Thereafter, the molecular structures are isolated by segmenting these masked sections within the document. However, a notable drawback emerges during mask expansion, where adjacent non-chemical sections of the molecular structures also get masked. This introduces a considerable amount of noise within the segmented molecular structures. In their evaluation, Rajan et al. curated a dataset of 25 articles from three distinct journal categories (*Molecules, Phytochemistry,* and *Journal of Natural Products*) to evaluate the efficacy of their model. Their analysis showed that, on average, ~ 11.2% of the segmented molecular structures contained noise, predominantly originating from non-chemical structures.

**Fig. 1:**
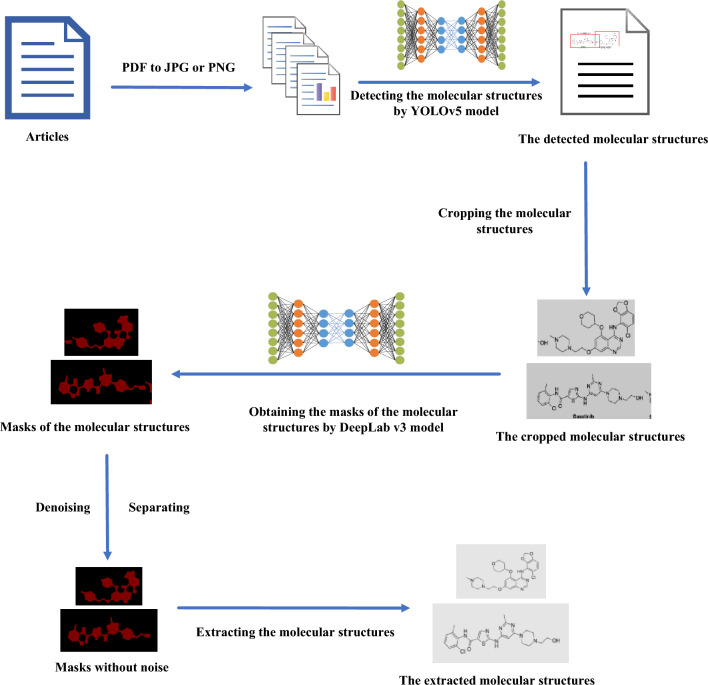
Graphical workflow summary for YoDe-Segmentation

In this study, we introduce a refined method for the automated, noise-free extraction of molecular structures, termed YoDe-Segmentation. This process is segmented into three primary phases. Initially, the molecular structure detection phase employs the YOLOv5 network model, specifically modified with a tailored prediction frame, to identify and subsequently crop molecular structures found within academic literature. The subsequent phase, referred to as the mask stage, utilises the DeepLabv3 network model to process these cropped molecular structure images, yielding a corresponding mask map of the molecular structure. The final phase encompasses denoising and differentiating the mask. Here, the molecular structure mask procured via the DeepLabv3 network model undergoes further refinement via an enhanced seed algorithm. This procedure results in the acquisition of a pristine molecular structure descriptor (as shown in Fig. 1).

### Molecular structure detection

During the molecular structure detection phase, we used the YOLOv5s model from the YOLOv5 network developed by Ultralytics [18]. Our computational environment operated on Python version 3.9, with PyTorch 1.6 serving as the deep learning framework [19].

To train the YOLOv5 network model, we curated a dataset sourced from papers published in the Journal of Medicinal Chemistry during 2010, 2011, and 2012. Randomly selected articles were converted into PNG image format via the Python Office package [20]. From this collection, a subset of 1933 images showing molecular structures was selected. The manual annotation of each molecular structure in the image was facilitated using the LabelImg tool [21]. Each molecular structure was meticulously enclosed within a singular rectangular boundary, with each boundary encompassing only one complete molecular structure. Cumulatively, 17,241 labelled regions were obtained. This collection of annotated images formed the training dataset for the YOLOv5 model. The dataset was divided into training and validation sets with 90% and 10% accuracy, respectively. The YOLOv5s model from the YOLOv5 network, complemented by pretrained models and preset hyperparameters supplied by Ultralytics, was employed. The model, set with a batch size of four, underwent training across 300 epochs on a robust computing server outfitted with an NVIDIA 1080Ti, 64 GB of RAM, and a 16-core Intel Core i7-11700 CPU.

**Fig. 2:**
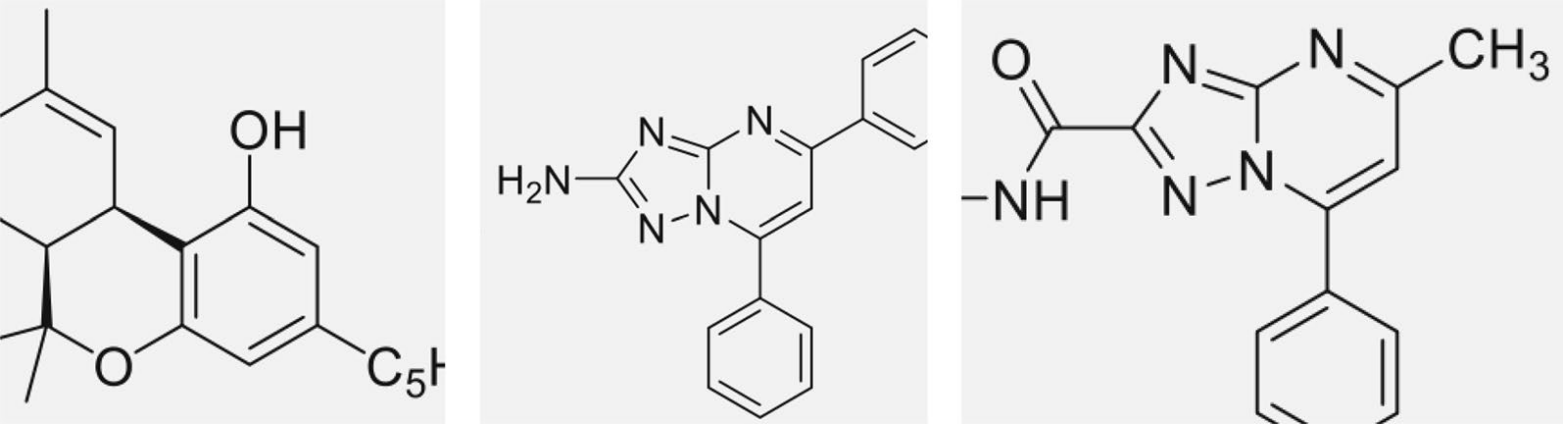
Images showing only incomplete chemical molecular structures

**Fig. 3:**
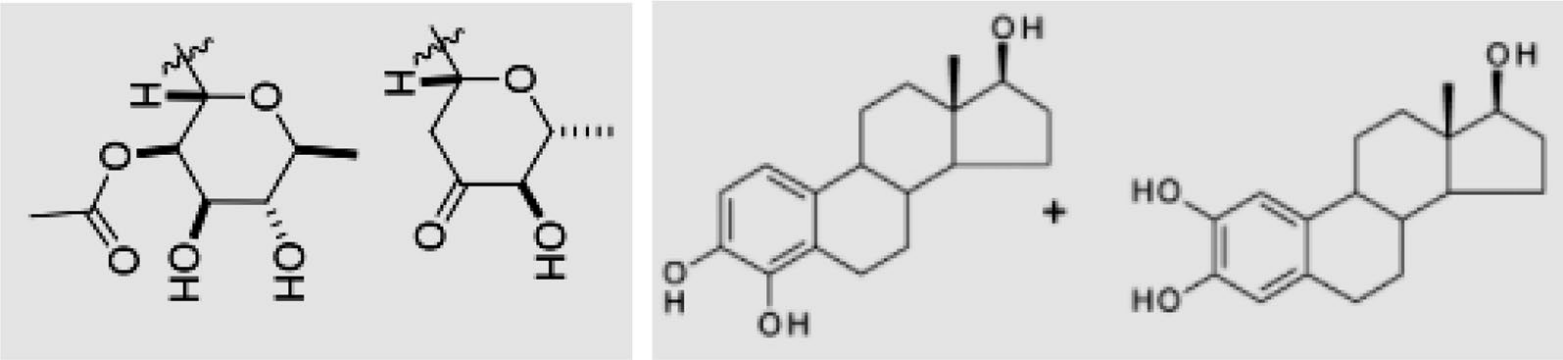
A prediction frame with multiple molecular structures

**Fig. 4:**
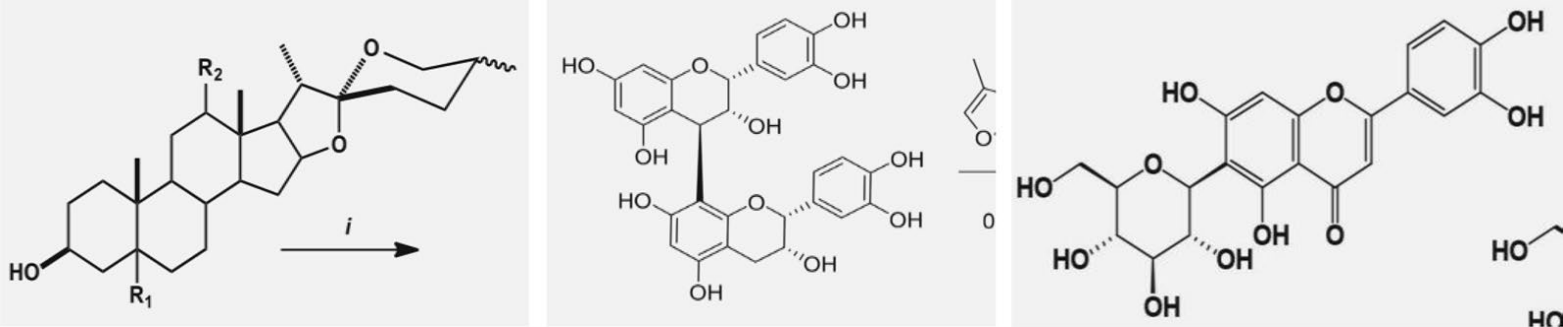
Images with both complete chemical molecular structures and non-chemical molecular structure elements

After training the YOLOv5 network model utilising the defined dataset and computational resources, we employed the model to identify and crop the molecular structure areas within the images. These cropped depictions essentially comprised two constituents: the integral molecular structure and accompanying noise. This preliminary output had three significant limitations:

1. Incompleteness of the cropped molecular structure images (Fig. 2).
2. Occasional occurrence of multiple molecular structures within a single predictive frame (Fig. 3).
3. The Presence of noise is delineated as the simultaneous inclusion of complete chemical molecular structures and unrelated non-chemical elements within the cropped images (Fig. 4).

To effectively address these limitations, we implemented solutions at distinct phases of the YoDe-Segmentation process. The initial issue was addressed in the current stage, while the subsequent issue was managed via precise preprocessing in the following stage and refined image processing in the final stage. The final issue was addressed by employing an advanced seed algorithm in the last stage.

Maintaining structural integrity is crucial for the seamless extraction of complete chemical molecular structures from scholarly literature. This necessitates the YOLOv5 model to recognise and crop the entire molecular structure during detection. To enhance the integrity of the structures identified, we broadened the predictive frame size facilitated by YOLOv5 to enable comprehensive detection of molecular structures. However, a minimally extended frame could result in incomplete molecular captures, while an overly expanded frame might contain additional non-chemical structural noise.

**Fig. 5:**
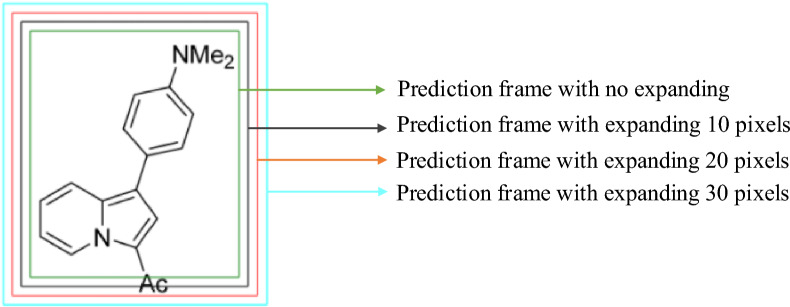
Example of different-sized prediction frames. Molecular structures are complete when expanded by 20 or 30 pixels but incomplete with a 10-pixel expansion or no expansion

Subsequently, we studied the impact of three distinct predictive frame expansions—10, 20, and 30 pixels—on a dataset consisting of 200 molecular structure-bearing images. These images were extracted from articles circulated in the *Journal of Medicinal Chemistry*, with those images from the initial training dataset being excluded. The results indicated that a 10-pixel augmentation ensured the completeness of 98.7% of the molecular structures, while 20 and 30 pixels increments ensured structural completeness. However, the 30-pixel enlargement presented a downside, introducing a higher noise quotient in the molecular structure imagery. Based on these experimental results, we set the expansion parameter of the molecular structure detection model at an optimal 20 pixels, as shown in Fig. 5.

### Obtaining the masks

**Fig. 6:**
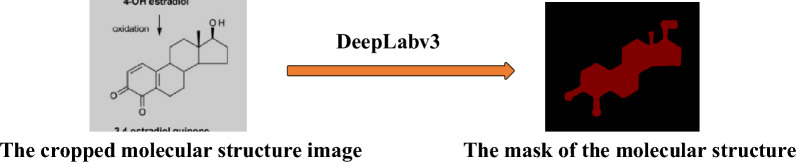
Use of the DeepLabv3 network model to process the cropped molecular structure image and generate the mask map

To address the second issue, we initially employed a semantic segmentation model to process cropped images, facilitating noise removal and attaining a molecular structure mask map. The DeepLabv3 semantic segmentation model [22], provided by Google, was instrumental in achieving this. Using the trained YOLOv5 model, we created a training dataset for DeepLabv3 comprising cropped images. The YOLOv5 model was deployed to detect and crop images derived from articles in the Journal of Medicinal Chemistry from 2010 to 2014. Subsequently, LabelMe [23], a tool designed for semantic segmentation annotation, was utilised to annotate the images randomly. This resulted in the labelling of 11,726 images demarcating molecular structures using polygons. We allocated 90% of the final dataset for training purposes and remaining 10% for validation. This trained model exhibited the capacity to mask components of molecular structures within cropped images, thereby generating the corresponding mask maps (Fig. 6).

### Denoising and separating the masks

**Fig. 7:**
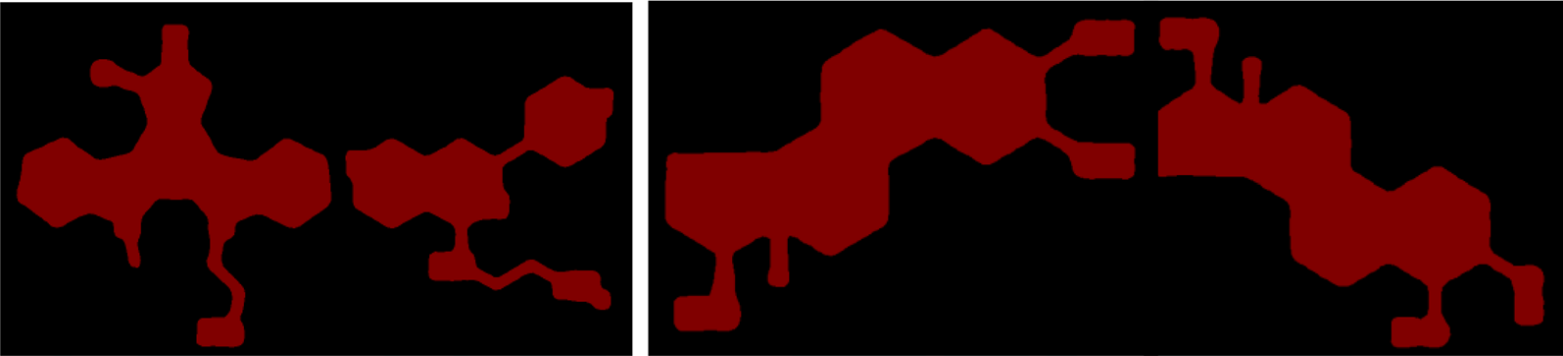
A prediction frame with multiple complete molecular structures

During the molecular structure identification phase, YOLOv5 demonstrated remarkable efficiency in recognising molecular structures within images. However, there were occurrences where the detection frame encompassed more than a single molecular structure, leading to the emergence of masks containing multiple comprehensive molecular structures during the molecular structure mask acquisition phase (Fig. 7). Given our objective of individually extracting each molecular structure so that every resultant image encapsulated a single molecular structure, it became imperative to manipulate the created mask maps. This manipulation involved transforming maps that contained several molecular structures into multiple distinct mask maps, each presenting a single molecular structure.

**Fig. 8:**
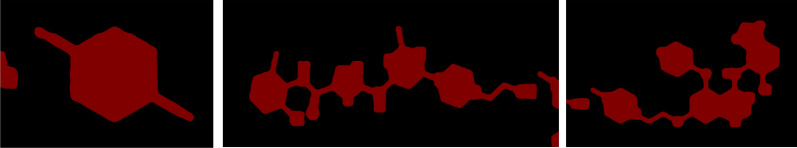
Mask maps with little noise

While securing molecular structure masks, DeepLabv3 could recognise the molecular structure; however, some noise was observed (Fig. 8). Consequently, a fraction of the produced mask maps contained noise. We addressed this issue by applying denoising and separation methodologies to the initially created mask maps.

The resulting mask map presented two exclusive pixel values: 0 and 128. The pixel value of 128 was provisionally assigned to serve as a molecular structure mask, effectively concealing noise. Following this, we employed a region-filling algorithm [24] to compute the pixel counts of each distinct mask. Among these, the mask with the maximal pixel count was discerned as the representative mask for the molecular structure.

To navigate the residual noise and concurrently address the challenge posed by prediction frames featuring multiple masks, we introduced a metric termed the pixel ratio (PR). This metric encapsulates the proportion of pixels in a given mask (denoted as p_mask_) relative to the pixels in the largest mask within the frame (p_largest mask_). Mathematically, the equation is:

$${\text{PR}} = \frac{{{\text{p}}_{{{\text{mask}}}} }}{{{\text{p}}_{{\text{largest mask}}} }}.$$

It was observed that noise masks typically had a pixel count distinctly smaller than that of the molecular structure masks. Conversely, within a singular prediction frame, molecular structure masks bore relatively comparable pixel counts. As a logical progression, we computed the PR for each mask. If the PR of a mask exceeded a specific threshold, it was deemed a genuine molecular structure mask; if it was below this threshold, it was designated as noise. We denoted this threshold as the pixel ratio threshold (PRT).

**Fig. 9:**
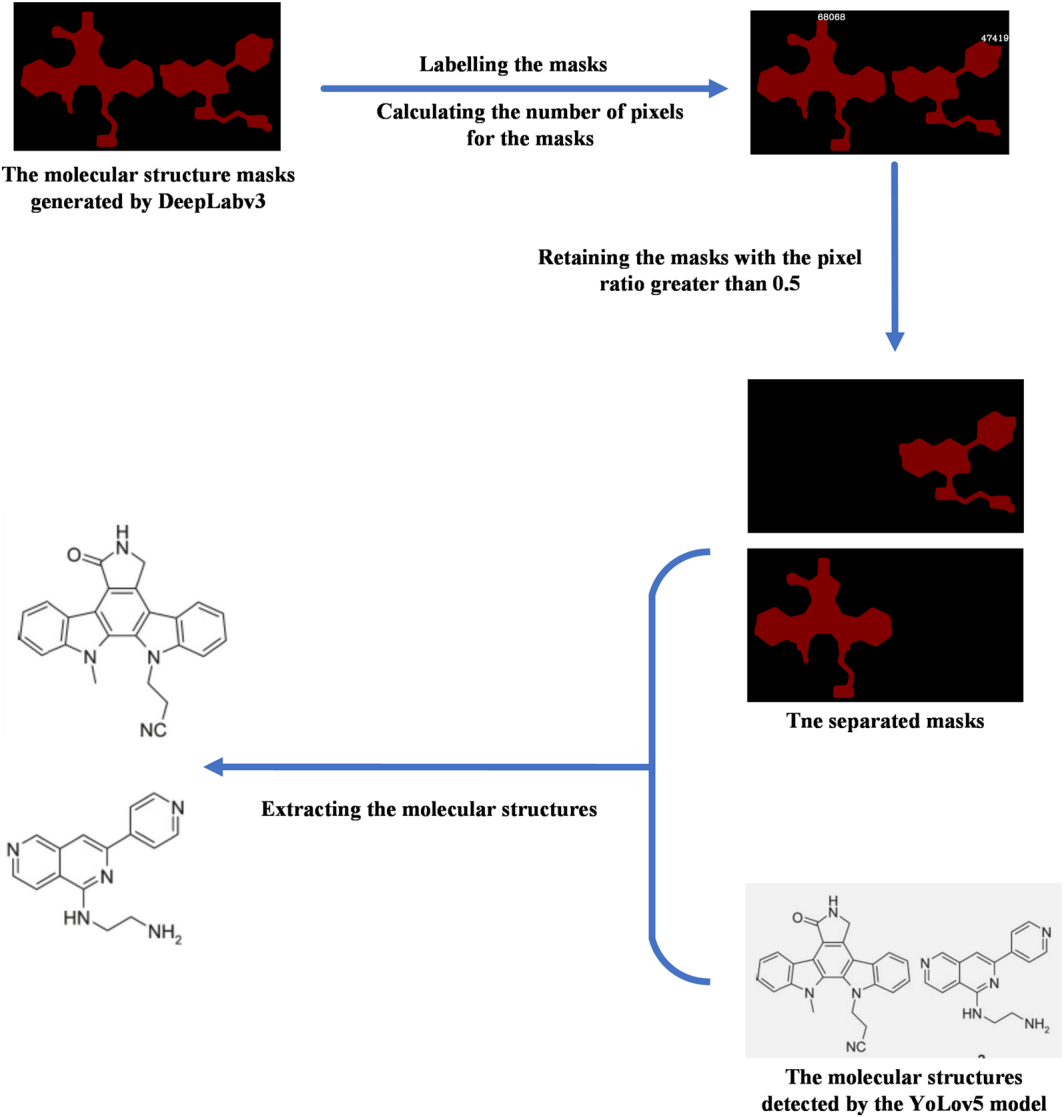
Separation example: The mask map has two complete molecular structure masks with pixel counts of 63,060 and 47,419. Their PR values are 1 and 0.696, respectively. Both exceed 0.5; therefore, the corresponding molecular structures are separated and extracted

**Fig. 10:**
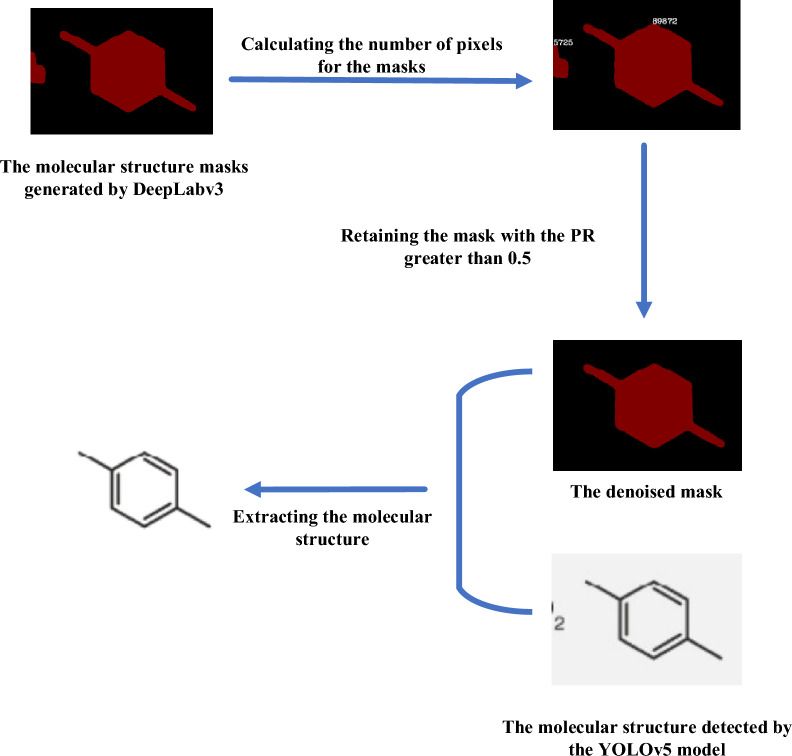
Denoising example: The mask map contains two masks with pixel counts of 89,872 and 5,725. Their PR values are 1 and 0.063, respectively. Only the molecular structure with a PR of 1 is extracted

To empirically ascertain the value of PRT, assessments were performed on 70 masks, each containing multiple molecular structures, along with 70 masks characterised by noise. An analysis of the PR for each of these revealed that genuine molecular structures had PR values ranging from 0.55 to 1. By contrast, the noise masks were situated between 0 and 0.2. Informed by these findings, we designated the PRT as 0.5. Consequently, masks that exhibited a PR surpassing 0.5 were classified as genuine molecular structure masks, as exemplified in Fig. 9. Those falling below the PRT of 0.5 were classified as noise masks, as illustrated in Fig. 10. The final step encompassed executing image operations on each authentic molecular structure mask, paired with its corresponding cropped image, to derive a comprehensive and pristine molecular structure description.

### Validation

To evaluate the efficiency of the YoDe-Segmentation process, we conducted an evaluation deploying a methodology akin to that utilised in DECIMER Segmentation assessments. While DECIMER Segmentation typically encompasses the random selection of 25 articles from journals like *Molecules*, *Phytochemistry*, and *Journal of Natural Products*, our strategy sought to augment both the diversity and volume of the validation dataset. We included articles from an additional journal—*Journal of Medicinal Chemistry*—and escalated the article tally to 100 for each journal, culminating in an aggregate of 400 articles. These articles were then distributed across four subsets for a meticulous evaluation, with each subset embracing 100 articles, evenly distributed with 25 from each journal, including a contingent derived from the DECIMER Segmentation’s assembly.

Subsequently, we transformed these 400 articles into 4549 PNG images utilising the Python Office package, resulting in groups with distinct image counts: 1229 in Group 1, 1059 in Group 2, 1089 in Group 3, and 1172 in Group 4. Manual scrutiny revealed the presence of 9140 molecular structures dispersed across the groups, with 2724 in Group 1, 2009 in Group 2, 2303 in Group 3, and 2104 in Group 4. The ensuing phase involved quantifying the totality of accurately extricated molecular structures through YoDe-Segmentation and determining the extraction efficacy for each group.

## Results and discussion

During the inaugural phase, which primarily focused on the detection of molecular structures, YoDe-Segmentation demonstrated a remarkable precision of 99.92%, identifying 9133 molecular structures and falling short by a mere seven structures distributed unevenly across the journals (1 in *Journal of Medicinal Chemistry*, 3 in *Journal of Natural Products*, 2 in *Phytochemistry*, and 1 in *Molecules*). Transitioning to the mask acquisition phase, we harnessed the capabilities of the DeepLabv3 model to craft masks corresponding to the molecular structures and fabricate an associated mask map. Despite encountering minimal noise or the simultaneous depiction of multiple molecular structures within a single mask image, the process continued to the denoising and segregating phases. Herein, we implemented an enhanced seed algorithm for the segregation and purification of the mask image, ultimately yielding a depiction devoid of noise. The culmination of this stage manifested in the procurement of a pristine molecular structure representation through meticulous image processing of the cropped molecular structure image.

**Fig. 11:**
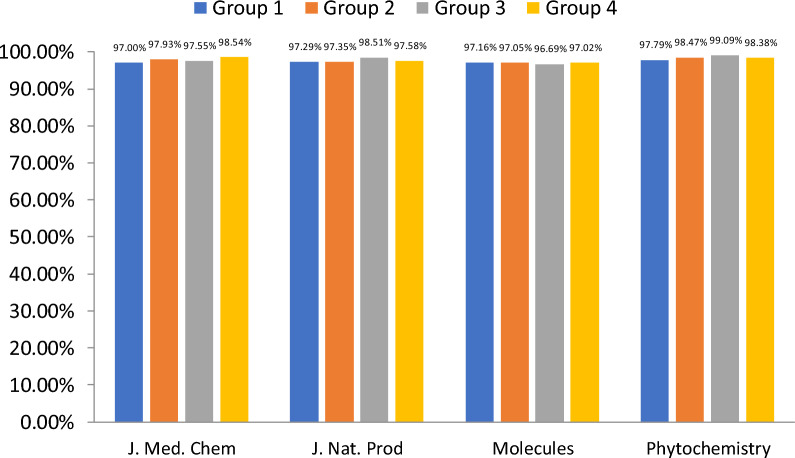
Overview of the YoDe-Segmentation validation results

YoDe-Segmentation supported the extraction of 97.62% molecular structures, with group-specific extraction rates of 97.17% for Group 1, 97.76% for Group 2, 97.65% for Group 3, and 98.05% for Group 4. A comprehensive breakdown of the performance metrics specific to each journal within the individual groups is shown in Fig. 11.

**Fig. 12:**
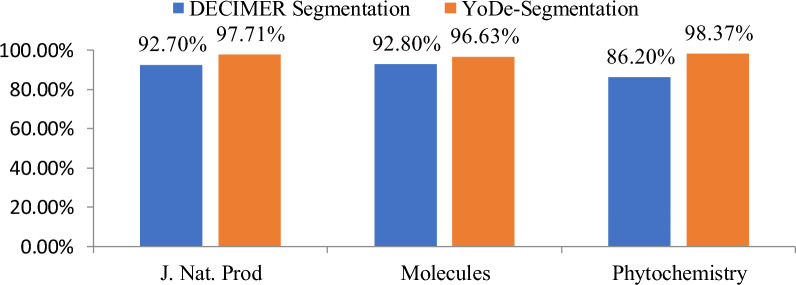
Comparison of evaluation results between YoDe-Segmentation and DECIMER segmentation in three journals

According to the evaluation results, YoDe-Segmentation performed excellently on articles sourced from the *Journal of Natural Products, Phytochemistry, and Molecules* despite the absence of training on articles from these specific journals. The average accuracy rate achieved by YoDe-Segmentation across these journals was 97.56%. Dissecting this further, the *Journal of Natural Products* registered an accuracy of 97.71%, Molecules of 96.95%, and *Phytochemistry* of 98.37%. When compared with the extraction metrics of DECIMER Segmentation, a discernible distinction emerges. The average extraction rate for DECIMER Segmentation across the three journals was 91.3%, with individual rates of 92.7% for the *Journal of Natural Products*, 92.8% for *Molecules*, and 86.3% for *Phytochemistry* (Fig. 12).

**Fig. 13:**
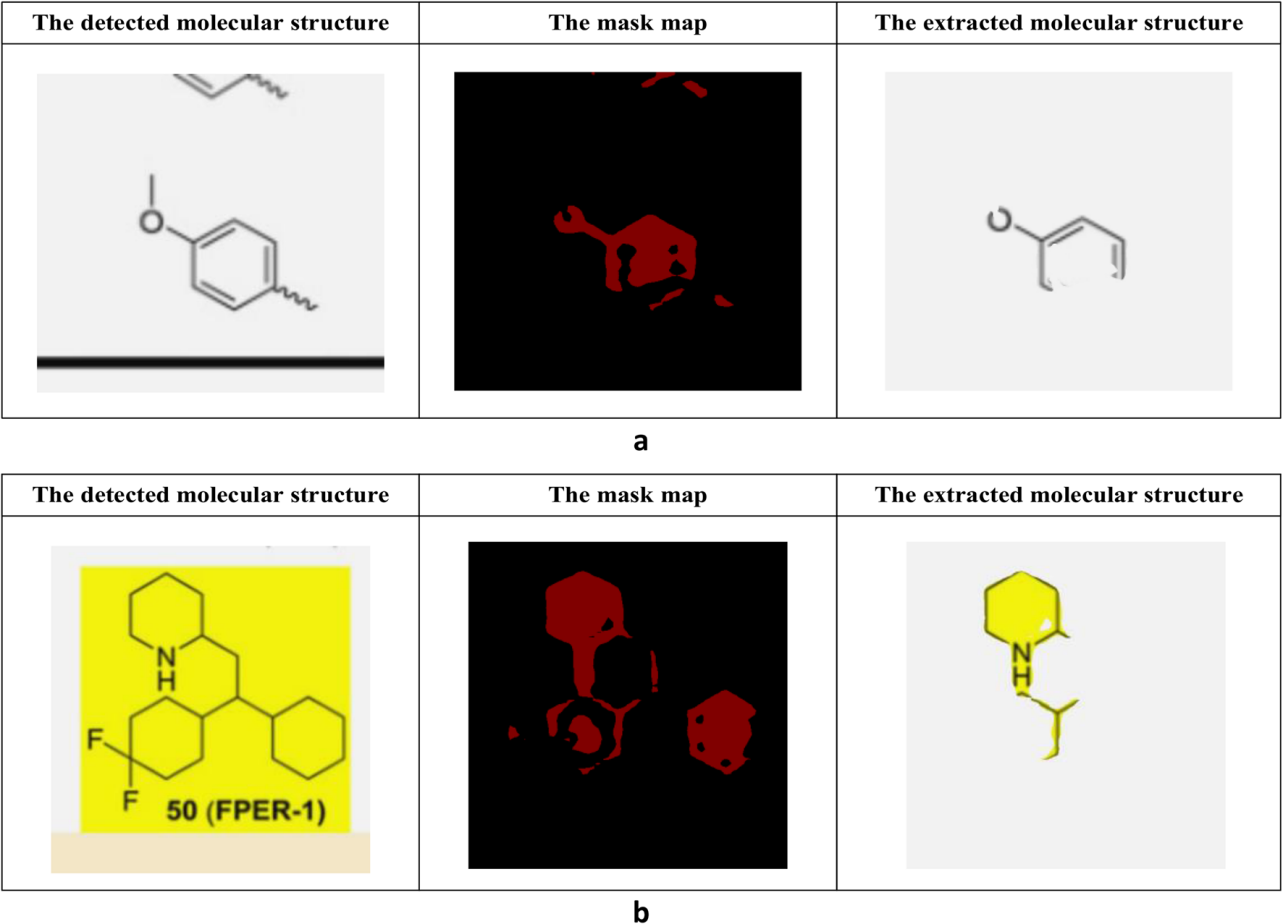
Extraction process for a low-resolution small molecule image (**a**). Extraction process of the molecular structure composed with coloured pixels (**b**)

Delving deeper into the molecular structure detection phase, YoDe-Segmentation showed a minor oversight, failing to detect 0.08% of molecular structures. As we transition to the segmentation and extraction processes, ~ 2.3% of molecular structures exhibited discrepancies, either manifesting as incomplete extractions or being interspersed with noise. A closer examination revealed that a predominant fraction of these discrepancies stemmed from incomplete extraction, with only 0.1% of the images being plagued with noise. An analytical deep dive of the undetected or fragmentarily extracted molecular structures unveiled certain trends: the majority of the overlooked structures were diminutive molecules. Simultaneously, incomplete extractions were primarily evident in the peripheral regions of molecular structures. Two recurring attributes surfaced in these aberrant images—a diminished resolution and the sporadic presence of coloured pixels within segments of the molecular structure, as shown in Fig. 13. An inherent limitation of the training datasets of YoDe-Segmentation was the paucity of images bearing these specific characteristics, leading to less-than-ideal outcomes when processing such images.

## Conclusion

In this study, we introduced YoDe-Segmentation, a new tool for extracting detailed molecular structures from scientific papers. Although we only used articles from the *Journal of Medicinal Chemistry* to train the tool, it also proved effective in handling articles from other journals. Impressively, it identified and extracted ~ 98% of the molecular structures present in our wide range of test documents, demonstrating both reliability and versatility.

However, the tool encountered challenges with certain types of images, particularly with the somewhat blurry images and those that lacked detail or contained coloured parts in the structures. These difficulties arose owing to the limited examples of such images in the training data. We are confident that adding our training data to include more of these types of images will enable YoDe-Segmentation to recognise and extract molecular structures from a wider variety of images.

In future studies, we will enrich our database with a greater variety of molecular structure images, including those with lower resolutions and coloured components. This enhancement will improve the performance of YoDe-Segmentation, ensuring its ability to extract high-quality molecular structure data reliably. This step is crucial for the next phase of our study, where we plan to develop methods for automatically translating these structures into specialised chemical notation systems like SMILES, SELFIES [25], and DeepSMILES [26]. This progression will refine the extraction process and pave the way for exciting advancements in the chemical research field.

## Data Availability

The dataset and source code supporting the conclusions of this study are available in the [YoDe-Segmentation] repository. [unique persistent identifier and hyperlink to the dataset are available at https://github.com/OneChorm/YoDe-Segmentation].

## Abbreviations

OCSR: Optical chemical structure recognition
OSRA: Optical structure recognition application
DECIMER: Deep learning for chemical image recognition
PNG: Portable network graphics
SMILES: Simplified molecular-input line-entry system
SELFIES: Self-referencing embedded strings
RAM: Random access memory
CPU: Central processing unit
CSR: Chemical schema resolver
CNN: Convolutional neural networks
PR: Pixel ratio
PRT: Pixel ratio threshold
